# Two-Week Interval Hypofractionated Stereotactic Radiosurgery for Benign Intracranial Tumors: Volumetric Kinetics and Radiobiological Rationale

**DOI:** 10.3390/cancers18040617

**Published:** 2026-02-13

**Authors:** Seung Woo Hong, Junhyung Kim, Jinu Rim, Jung Woo Yu, Hyun Ho Jung, Jong Hee Chang, Won Hee Lee, Won Seok Chang

**Affiliations:** 1 Department of Neurosurgery, Hanyang University Seoul Hospital, Hanyang University College of Medicine, Seoul 04763, Republic of Korea; hsw0777@hyumc.com; 2 Department of Neurosurgery, Yonsei Gamma Knife Center, Severance Hospital, Yonsei University College of Medicine, Seoul 03722, Republic of Korea; jkim1518@yuhs.ac (J.K.); hotearsns@yuhs.ac (J.R.); trajectoryofstar@yuhs.ac (J.W.Y.); junghh@yuhs.ac (H.H.J.); changjh@yuhs.ac (J.H.C.); 3 Department of Neurosurgery, Busan Paik Hospital, School of Medicine, Inje University, Busan 47391, Republic of Korea

**Keywords:** radiosurgery, benign neoplasm, pituitary adenoma, acoustic neuroma, radiobiology

## Abstract

Background/Objectives: Hypofractionated stereotactic radiosurgery (hfSRS) is increasingly used for benign intracranial tumors that are large or located near critical neural structures to reduce treatment-related toxicity. However, the optimal interval between fractions remains poorly defined, particularly for slowly proliferating benign tumors. This study evaluated clinical outcomes and longitudinal volumetric response patterns following Gamma Knife hfSRS delivered at fixed two-week intervals, with particular attention to the biological relevance of fraction timing. Methods: We retrospectively analyzed 126 patients with benign intracranial tumors, including meningioma, non-functioning pituitary neuroendocrine tumor (PitNET), vestibular schwannoma, and craniopharyngioma, treated between 2016 and 2022. Treatment was delivered in 2–5 fractions at fixed two-week intervals using Gamma Knife radiosurgery. Radiological outcomes included tumor control rate and longitudinal volumetric changes, while clinical outcomes included visual, auditory, and endocrine function. Propensity score matching was performed in PitNET and vestibular schwannoma cohorts to compare hfSRS with single-fraction stereotactic radiosurgery while minimizing baseline imbalances. Results: The overall tumor control rate was 98.4%. Across the entire cohort, tumors demonstrated a median volume reduction of −0.64% per month. In the propensity score–matched PitNET cohort, tumor control was comparable between treatment groups, whereas hfSRS was associated with earlier and greater volumetric reduction over time compared with single-fraction treatment. In the matched vestibular schwannoma cohort, long-term tumor control was similar between groups; however, transient tumor enlargement occurred more frequently after hfSRS without adversely affecting long-term tumor control or functional hearing outcomes. Conclusions: Hypofractionated stereotactic radiosurgery delivered at fixed two-week intervals achieved excellent tumor control with acceptable toxicity in selected benign intracranial tumors. These findings support the clinical feasibility of a fixed two-week inter-fraction interval and suggest that fraction timing may represent a biologically relevant treatment parameter influencing early volumetric response patterns without compromising long-term outcomes.

## Simple Summary

Benign brain tumors often grow slowly but can cause serious problems when they are located near critical structures such as the optic nerves, brainstem, or hearing pathways. Stereotactic radiosurgery is a common non-surgical treatment, but delivering the full radiation dose in a single session may increase the risk of side effects in these sensitive areas. Dividing the treatment into several sessions, called hypofractionated radiosurgery, is therefore increasingly used, yet the best timing between sessions remains unclear. In this study, we evaluated a treatment approach in which radiation was delivered at fixed two-week intervals. We analyzed tumor size changes over time and clinical outcomes in patients with common benign brain tumors. Our findings show that this schedule achieved excellent tumor control with acceptable side effects and produced distinct but predictable patterns of tumor response. These results suggest that the interval between treatment sessions itself is an important factor in optimizing radiosurgery for benign brain tumors.

## 1. Introduction

Stereotactic radiosurgery (SRS) is an established treatment modality for a variety of intracranial tumors, providing excellent local control with minimal invasiveness. However, single-fraction SRS carries an inherent risk of radiation-induced complications, particularly when treating large lesions adjacent to critical structures such as the brainstem, optic apparatus, or cochlea. For example, when the normal brain volume receiving more than 12 Gy (V12) exceeds 10–15 cm^3^ in a single session, the risk of radiation necrosis increases significantly [1,2].

Hypofractionated stereotactic radiosurgery (hfSRS), typically delivered in 3–5 fractions, has emerged as an effective strategy to mitigate these risks. In malignant tumors, hfSRS offers radiobiological advantages by fractionating the dose: it maintains an equivalent tumor control probability (given an α/β of approximately 10 Gy for tumor tissue in biologically effective dose (BED) calculations) while reducing the BED to late-responding normal tissues (with an α/β of approximately 2–3 Gy) [3]. This approach is particularly beneficial in lesions adjacent to low α/β structures such as the brainstem, optic apparatus, and cranial nerves, reducing the risks of radiation-related toxicity and necrosis.

For intracranial benign tumors, which share a similar α/β ratio with surrounding normal tissues, fractionation does not confer the radiobiological benefit of selectively sparing late-responding tissues. Nonetheless, it may provide dosimetric advantages by mitigating dose hotspots and improving dose homogeneity in larger lesions [4,5].

Another key consideration in hfSRS is the optimal interval between fractions. For rapidly proliferating malignant tumors, short intervals of 48–72 h are preferred to maximize tumor control and limit repopulation. However, some centers have adopted longer schedules, such as once weekly or biweekly treatments, in order to reduce sublethal damage to adjacent normal tissues [3,6]. Such long-interval approaches may be particularly advantageous for benign tumors, where rapid repopulation is less relevant but preservation of surrounding neural function is critical. Nevertheless, the clinical and biological implications of inter-fraction interval selection remain poorly defined, particularly for slowly proliferating tumors in which classical malignant tumor paradigms may not apply.

The present study aims to systematically evaluate the clinical rationale and treatment outcomes of hfSRS delivered at fixed two-week intervals. Unlike most previous investigations that primarily focused on malignant tumors, this study emphasizes early radiological responses and functional preservation in patients with benign intracranial tumors. Importantly, few studies have examined the inter-fraction interval itself as an independent biological variable in hfSRS for benign intracranial tumors.

## 2. Materials and Methods

### 2.1. Study Design and Ethical Approval

This retrospective cohort study was performed using a prospectively collected database of patients with benign intracranial tumors who underwent Gamma Knife hfSRS at our institution between March 2016 and February 2022.

### 2.2. Patient Selection

During this period, a total of 157 patients received hfSRS for benign intracranial tumors. Patients with functioning pituitary neuroendocrine tumor (*n* = 19), hemangioma (*n* = 3), or pituitary oncocytoma (*n* = 2) were excluded. In addition, patients with follow-up shorter than 36 months or with loss of radiological follow-up were excluded (*n* = 7). The final study cohort therefore comprised 126 patients: 32 meningiomas, 34 non-functioning pituitary neuroendocrine tumors (NF-PitNET), 49 vestibular schwannomas (VS), and 11 craniopharyngiomas (Figure S1). Meningiomas included in this study were limited to WHO grade 1 tumors. WHO grade 2 and 3 meningiomas were excluded from the analysis. 

### 2.3. Study Objectives

The aim of the study was twofold. First, we sought to evaluate early- to mid-term radiological and clinical outcomes of hfSRS in the four most common benign intracranial tumors. Second, we compared hfSRS with single-fraction stereotactic radiosurgery (sfSRS) in patients with NF-PitNET and VS using propensity score matching (PSM) with patients who underwent single-fraction SRS for NF-PitNETor VS at our institution in the same period.

### 2.4. Preradiosurgical Evaluation

The Snellen visual acuity test and Humphrey visual field test were administered to patients with tumors in proximity to the anterior optic pathway (AOP). For patients with pituitary lesions, a basal hormone test (BHT) was obtained. In cases with tumors involving the internal auditory canal, a pure tone audiometry (PTA) test was performed to evaluate preradiosurgical hearing function.

### 2.5. Radiosurgery Techniques

Gamma Knife radiosurgery was primarily delivered using a frameless thermoplastic mask–based technique, with frame-based fixation reserved for patients unsuitable for mask immobilization. High-resolution contrast-enhanced MRI was obtained within 24 h prior to treatment and used for target delineation and dose planning. All plans were generated using Leksell GammaPlan (version 11.1.0) and delivered with the Gamma Knife Icon system (Elekta AB, Stockholm, Sweden) by experienced neurosurgeons in collaboration with a medical physicist.

Hypofractionated stereotactic radiosurgery was delivered in 2–5 fractions at fixed two-week intervals, with three fractions used in the vast majority of patients (120 of 126, 95.2%). Two-fraction or five-fraction schedules were reserved for selected cases requiring either dose escalation or additional per-fraction dose reduction because of tumor size or proximity to critical organs at risk.

Dose prescription was guided by the linear–quadratic (LQ) biologically effective dose (BED) model, nd[1 + d/(α/β)], with the intent of achieving biological equivalence (±5%) to a conventional single-fraction marginal dose of approximately 13–14 Gy, which represents the standard prescription range for benign intracranial tumors at our institution. An α/β ratio of 3 was adopted solely as a pragmatic planning assumption to ensure internal dose normalization within the LQ framework, rather than to imply tumor-selective radiobiological effects, given the known limitations of the LQ model at high-dose ranges.

Under this scheme, fraction doses typically ranged from 5.0 to 7.0 Gy per fraction, depending on the total number of fractions, tumor characteristics, and organ-at-risk constraints. Across the entire cohort, hypofractionated stereotactic radiosurgery was delivered predominantly in three fractions (120 of 126 patients, 95.2%). Two-fraction or five-fraction schedules were selectively employed in a small subset of patients to allow either dose escalation or additional per-fraction dose reduction based on tumor size and proximity to critical organs at risk. The median prescribed marginal dose per fraction was within the range of 5.0–7.0 Gy, corresponding to a biologically effective dose intentionally matched (±5%) to a conventional single-fraction prescription of approximately 13–14 Gy.

For organs at risk, including the optic apparatus and cochlea, established single-fraction dose constraints (10 Gy for the optic pathway and 6 Gy for the cochlea) were converted into fraction-specific maximum dose limits using an α/β ratio of 2. These converted constraints directly informed final dose selection and rounding (0.1–0.5 Gy increments), prioritizing organ-at-risk protection while maintaining steep dose gradients.

Representative dose conversions and corresponding organ-at-risk constraints are summarized in Supplementary Table S1.

HfSRS was preferentially selected for tumors with relatively large volumes, lesions abutting or compressing critical neural structures, or cases in which single-fraction dose constraints were anticipated to limit treatment feasibility. In selected patients, hypofractionation was additionally considered to facilitate functional preservation when the therapeutic margin between tumor control and normal tissue tolerance was narrow.

### 2.6. Clinical and Radiological Follow-Up

Patients visited the outpatient clinic and were evaluated for any clinical symptoms at 1, 3, 6, and 12 months after the treatment, and every 12 months thereafter. Prophylactic corticosteroids were not routinely administered; however, short-term dexamethasone (typically 2–4 mg/day for several days) was prescribed at the discretion of the treating physician in cases with significant peritumoral edema or new-onset neurological symptoms following SRS.

Radiological outcomes were assessed via MRI at 6 and 12 months after the radiosurgery, and every 12 months thereafter. In patients with PA, BHT was performed to observe hormonal changes after radiosurgery, and patients who complained of hearing deterioration and visual symptoms underwent PTA tests and ophthalmologic examinations, respectively.

Tumor volumetric measurements were derived from serial contrast-enhanced MRI using a standardized semi-automated segmentation workflow. All segmentations were performed by a single experienced neurosurgeon using identical software settings across all time points to ensure longitudinal consistency and minimize intra-study measurement variability.

Inter-fraction volumetric changes between individual hypofractionated sessions were not systematically analyzed in this study. Although interval imaging was occasionally performed during early protocol implementation—particularly in patients with relatively large tumors—no clinically meaningful volumetric or morphological changes were observed that necessitated modification of target delineation or dose planning. Accordingly, treatment planning throughout the hypofractionated course was based on the initial preradiosurgical imaging.

### 2.7. Outcome Assessment

Volumetric measurements were obtained as described above (Section 2.6) and were used to define radiologic endpoints.

The primary radiological endpoint was the tumor control rate (TCR), assessed using serial volumetric measurements on MRI. Tumor control was defined according to a RANO (Response Assessment in Neuro-Oncology)-based radiologic framework, in which complete response, partial response, and stable disease were collectively considered controlled disease, and progressive disease was defined as sustained volumetric enlargement on serial follow-up imaging.

Volumetric tumor response was evaluated using two complementary metrics.

First, overall volumetric kinetics were descriptively summarized using percentage volume change per month across the entire cohort.

Second, for comparative analyses, percentage volume change from baseline at predefined follow-up time points (12, 24, and 36 months) was used to ensure uniform temporal comparison between treatment groups.

The final percentage volume change from baseline at the last radiologic follow-up was additionally used as a summary measure of cumulative tumor response.

Pseudoprogression was operationally defined as a transient increase in tumor volume exceeding measurement variability (>10%), followed by subsequent stabilization or regression on serial imaging without additional treatment [7].

Radiation-induced optic neuropathy (RION) was diagnosed when deterioration in visual acuity or visual fields was documented. Visual acuity decline was defined as a decrease in one or more lines on the Snellen chart, and visual field defects as ≥10% loss on Humphrey perimetry. Vascular compromise was identified as ≥30% luminal narrowing on MRI. Facial nerve palsy was graded according to the House–Brackmann classification. Functional hearing loss was defined as deterioration from preradiosurgical serviceable hearing—Gardner-Robertson class I or II (pure tone average ≤50 dB and/or speech discrimination ≥50%)—to class III or worse (pure tone average >50 dB and/or speech discrimination <50%). New-onset hypopituitarism was diagnosed if patients with previously normal pituitary function required hormone replacement therapy after SRS.

### 2.8. Statistical Analysis

All statistical analyses were performed using IBM SPSS Statistics version 28.0 (IBM, Armonk, NY, USA). To compare treatment outcomes between hypofractionated stereotactic radiosurgery (hfSRS) and single-fraction stereotactic radiosurgery (sfSRS), propensity score matching (PSM) was conducted in patients with PitNET and vestibular schwannoma (VS).

Meningiomas were excluded from PSM because anatomical location substantially influences radiosurgical planning and outcomes. Skull base meningiomas are more likely to require lower marginal doses due to proximity to the optic apparatus and brainstem, whereas convexity or falcine meningiomas often allow higher marginal doses, leading to site-dependent differences in dosimetry and toxicity profiles [8,9]. Although stratification by anatomical site could theoretically address this confounding, subdividing the meningioma cohort by location would result in insufficient subgroup sizes, limiting the reliability and interpretability of a matched analysis. Therefore, meningiomas were analyzed descriptively rather than included in the PSM framework.

Propensity score matching was performed using baseline demographic and clinical variables, with additional tumor-specific covariates included to further reduce residual confounding. These tumor-specific covariates included Knosp grade for PitNET, as well as baseline hearing status and the presence of cystic components for vestibular schwannomas. Follow-up duration was excluded from the propensity model, as it does not represent a baseline factor at treatment initiation; instead, radiological outcomes were evaluated within a predefined and uniform analysis window.

To further reduce residual confounding, tumor-specific covariates were additionally included. For PitNET, cavernous sinus invasion was incorporated using a dichotomized Knosp classification (grades 0–2 vs. 3–4), reflecting differences in surgical resectability and radiosurgical planning. For VS, baseline hearing status was classified dichotomously as serviceable or non-serviceable, and cystic tumor composition was categorized as solid versus mixed/cystic, acknowledging the difficulty of precise volumetric quantification of cystic components and their potential influence on post-radiosurgical volumetric kinetics.

Patients were matched in a 1:1 ratio using a nearest-neighbor algorithm with a caliper width of 0.2 standard deviations of the logit of the propensity score, without replacement. Post-matching balance between groups was assessed using standardized mean differences. Categorical variables were compared using Fisher’s exact test or chi-square test, and continuous variables were analyzed using the Mann–Whitney U test or Student’s *t*-test, as appropriate. A two-sided *p*-value < 0.05 was considered statistically significant.

## 3. Results

### 3.1. Patient and Treatment Characteristics

A total of 126 patients were included in the study. SRS was administered as the primary treatment in 63 patients. The most common histology was schwannoma (*n* = 49), comprising vestibular schwannoma (VS, *n* = 46), trigeminal schwannoma (*n* = 2), and orbital schwannoma (*n* = 1). All NF-PitNET cases (*n* = 34) and craniopharyngiomas (*n* = 11) were treated with SRS as adjuvant therapy for residual or recurrent disease following surgical resection. Frameless SRS was the predominant modality, performed in 114 of 126 patients. Given the biological and clinical heterogeneity of benign intracranial tumors, detailed comparative analyses were intentionally focused on PitNET and vestibular schwannoma, which constituted the majority of the cohort and allowed robust volumetric and functional evaluation.

Fractionation schedules included two fractions in four patients, three fractions in 120 patients, and five fractions in two patients. Patient and treatment characteristics are summarized in Table 1.

### 3.2. Overall Outcomes

According to the RANO-based radiologic criteria, the overall tumor control rate—defined as the absence of progressive disease—was 98.4% (124/126). Two patients developed radiographic tumor progression during follow-up; both cases occurred in patients with meningioma. Neither patient required salvage treatment during the observation period, as progression was slow and not associated with clinical symptoms.

Volumetric tumor response was primarily evaluated using percentage volume change from baseline at predefined follow-up time points. At final radiologic follow-up, all tumor types demonstrated net volumetric reduction, with histology-specific response patterns summarized in Table 2. Across the entire cohort, tumors showed a net volumetric regression over time, with a median percentage volume change of −0.64% per month. Longitudinal assessment at predefined follow-up time points demonstrated histology-specific volumetric kinetics, with relatively monotonic shrinkage in meningioma, PitNET, and craniopharyngioma, and more dynamic early fluctuations in vestibular schwannoma. These overall volumetric trends provide descriptive context for the cohort as a whole, whereas tumor-specific comparative analyses were subsequently focused on PitNET and vestibular schwannoma.

Similar volumetric regression patterns were observed when the percentage volume change from baseline at last follow-up was analyzed. Volumetric kinetics differed by tumor type, with meningioma, PitNET, and craniopharyngioma showing relatively uniform volume reduction over time, whereas vestibular schwannoma exhibited greater variability in early post-treatment changes, including transient tumor enlargement.

Radiation-induced edema was observed in three patients (two with meningioma and one with VS), all of whom were asymptomatic and resolved spontaneously. Hydrocephalus developed in two VS patients, both requiring later shunt surgery. Radiation-induced vascular compromise of the internal carotid artery was identified in three meningioma patients with pre-existing tumor-related arterial narrowing. All cases were asymptomatic and managed conservatively with antiplatelet therapy after consultation with a cerebrovascular specialist.

Among 51 patients with cerebellopontine angle tumors, one patient (2.0%) experienced worsening of facial palsy. Of the 39 patients who had serviceable hearing at baseline, 16 (41.0%) developed functional hearing loss during follow-up.

Radiation-induced optic neuropathy (RION), defined as new-onset visual acuity or visual field deterioration after treatment, occurred in two patients (2.8%), none of whom required additional intervention. Among 40 patients with normal baseline pituitary function, two (5.0%) required initiation of hormone replacement therapy after SRS.

The overall radiological and clinical outcomes are summarized in Table 2.

### 3.3. Propensity Score Matching Analysis of hfSRS and sfSRS for PA and VS

PitNET and vestibular schwannoma were selected for propensity score–matched comparison with single-fraction SRS, given their relative homogeneity and sufficient sample size. After 1:1 propensity score matching in the PitNET cohort, baseline characteristics between the hypofractionated SRS and single-fraction SRS groups were well balanced. Detailed baseline characteristics before and after matching, along with standardized mean differences, are provided in Supplementary Table S2. Patient outcomes after matching for PitNET and vestibular schwannoma are summarized in Table 3.

**Figure 1:**
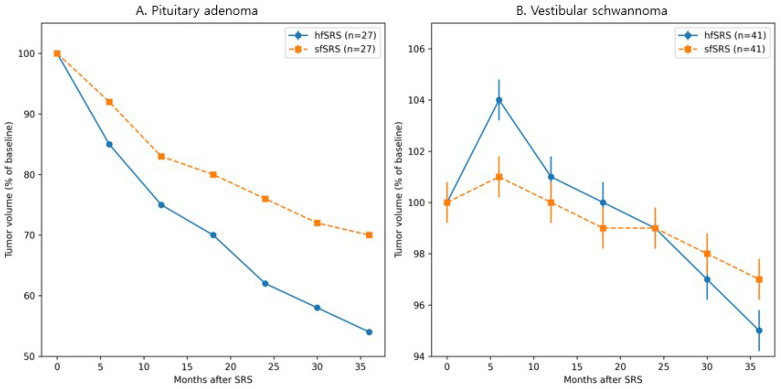
Longitudinal volumetric changes after stereotactic radiosurgery in propensity score–matched cohorts. (**A**) pituitary neuroendocrine tumor patients treated with hypofractionated Gamma Knife radiosurgery (hfGKRS) versus single-fraction Gamma Knife radiosurgery (sfGKRS). (**B**) Vestibular schwannoma patients treated with hfGKRS versus sfGKRS. Tumor volume is expressed as a percentage of baseline volume at each follow-up time point.

In the propensity score–matched PitNET cohort, tumor control rates were comparable between the hfSRS and sfSRS groups (96.3% vs. 100%, *p* = 1.00, Fisher’s exact test). Percentage volume change from baseline, assessed at fixed follow-up time points, demonstrated a greater degree of tumor regression in the hfSRS group. At 36 months after treatment, the estimated median percentage volume change was approximately −25% in the hfSRS cohort compared with −10% in the sfSRS cohort, indicating more pronounced volumetric reduction following hypofractionated treatment. Similar trends were observed when the percentage volume change at the last follow-up was analyzed. The incidence of radiation-induced optic neuropathy and new-onset hypopituitarism did not differ significantly between treatment groups (3.7% vs. 7.4%, *p* = 0.55). Figure 1A illustrates the longitudinal volumetric response patterns in the matched PitNET cohorts.

In the vestibular schwannoma cohort, propensity score matching achieved acceptable balance across most covariates; baseline characteristics before and after matching are summarized in Supplementary Table S3. Tumor control rates were comparable between the hfSRS and sfSRS groups. Percentage volume change from baseline, assessed at fixed follow-up time points, revealed distinct temporal response patterns between treatment groups. At 12 months after treatment, the hfSRS group more frequently exhibited transient tumor enlargement, whereas volumetric changes in the sfSRS group were more stable during early follow-up. At 36 months, both groups demonstrated net volumetric regression, with estimated median percentage volume changes of approximately −8% in the hfSRS group and −6% in the sfSRS group.

Pseudoprogression occurred significantly more often following hfSRS than sfSRS (41.5% vs. 19.5%, *p* = 0.03); however, long-term tumor control remained comparable between groups. The proportion of patients with serviceable hearing at baseline tended to be higher in the hfSRS group (73.2% vs. 56.1%, *p* = 0.10), although the incidence of functional hearing loss during follow-up did not differ significantly (40.0% vs. 60.9%, *p* = 0.17). Figure 1B illustrates the longitudinal tumor volume trajectories in the matched vestibular schwannoma cohorts. No patient required salvage treatment solely due to transient volumetric enlargement. Despite propensity score matching, a mild residual imbalance in baseline hearing status remained, reflecting the clinical preference for hypofractionation in patients with serviceable hearing; this variable was therefore interpreted with caution.

### 3.4. Illustrative Cases

#### 3.4.1. Case 1 (Figure 2)

**Figure 2:**
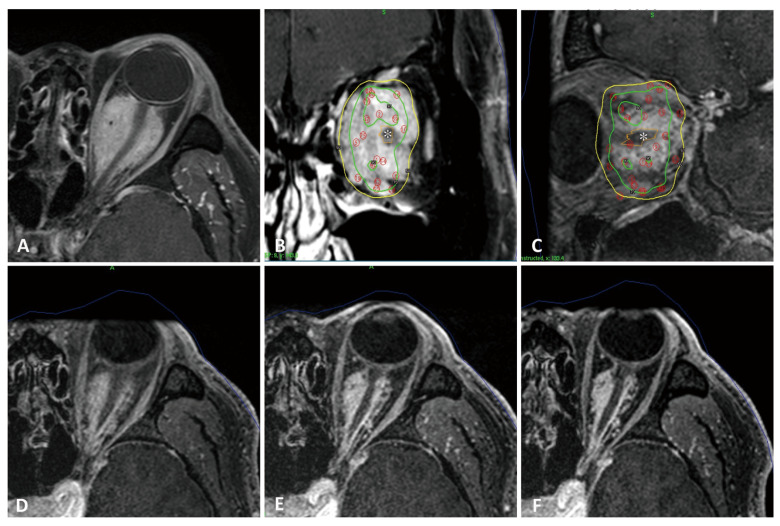
(**A**) Axial preradiosurgical MRI showing a meningioma encasing the left optic nerve. (**B**,**C**) Coronal (**B**) and sagittal (**C**) views of Gamma Knife dose planning; the optic nerve (white asterisk) is excluded from the 90% isodose line. (**D**–**F**) Follow-up MRIs at 6 months (**D**), 12 months (**E**), and 24 months (**F**) demonstrate marked tumor shrinkage.

A 58-year-old male presented with progressive proptosis and decreased visual acuity. Imaging revealed an intraorbital meningioma encasing the left optic nerve, with an initial volume of 12.6 cm^3^ (Figure 2A). The patient underwent three-fraction SRS, with a total dose equivalent of 13 Gy prescribed to the 50% isodose line at two-week intervals. The tumor volume decreased rapidly, measuring 4.8 cm^3^ at 6 months, 4.1 cm^3^ at 12 months, and 3.8 cm^3^ at 24 months post-treatment (Figure 2D–F). No RION was observed.

#### 3.4.2. Case 2 (Figure 3)

**Figure 3:**
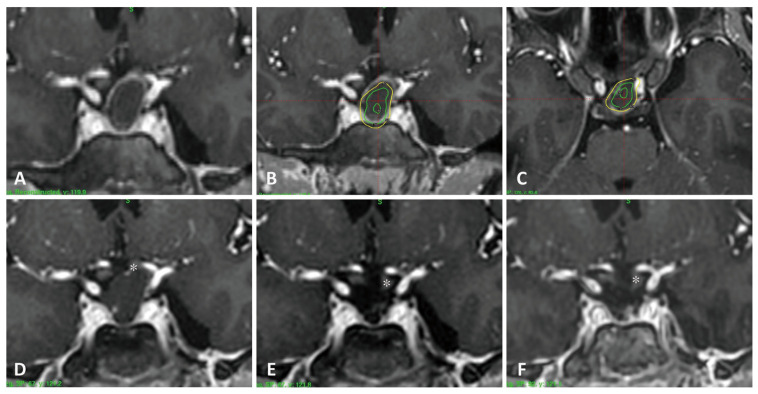
(**A**) Coronal preradiosurgical MRI showing tumor compressing the left optic nerve. (**B**,**C**) Coronal (**B**) and axial (**C**) dose-planning images. (**D**–**F**) Follow-up MRIs at 6 months (**D**), 12 months (**E**), and 24 months (**F**) demonstrate marked tumor shrinkage and decompression of the left optic nerve (white asterisk).

A 13-year-old boy underwent treatment for recurrent craniopharyngioma, presenting with an initial tumor volume of 1.6 cm^3^. Although no visual field deficit was detected, the tumor displaced the left optic nerve superiorly (Figure 3A). He received three-fraction SRS, equivalent to 13 Gy at the 50% isodose line, delivered at two-week intervals. Tumor volume decreased slightly to 1.4 cm^3^ at 6 months and was further reduced to 0.82 cm^3^ at 12 months and 0.75 cm^3^ at 24 months (Figure 3D–F). The patient remained free of visual symptoms.

## 4. Discussion

### 4.1. Overall Summary

Although hfSRS has been increasingly used for large or critically located intracranial tumors [3,10], most prior studies have primarily focused on fraction number or dose equivalence, with limited attention to the role of the inter-fraction interval itself. In particular, hypofractionation schedules have typically employed very short intervals (≤72 h) or weekly treatments, largely extrapolated from malignant tumor paradigms rather than being biologically optimized for benign disease [3,11].

This study differs from prior investigations by explicitly treating the inter-fraction interval itself as a biologically relevant variable, rather than a purely logistical parameter. Given the slow proliferative kinetics of benign tumors, suppression of rapid repopulation is less critical, whereas avoidance of radiation-induced edema and preservation of surrounding neural structures are paramount. By incorporating longitudinal volumetric analysis and propensity score–matched comparisons with single-fraction SRS, this study provides a nuanced assessment of how fraction timing influences early-to-mid-term volumetric kinetics, including earlier and greater percentage volume reduction over time in PitNET and increased but transient pseudoprogression in vestibular schwannoma.

Importantly, this approach is conceptually distinct from dose-staged radiosurgery, in which treatment sessions are separated by several months to allow edema stabilization before additional irradiation [12]. Instead, our protocol completes hypofractionated treatment within a biologically informed window prior to peak vasogenic edema, an approach we describe as a pre-edema completion strategy. Using this framework, hypofractionated SRS delivered at a fixed two-week interval was associated with favorable tumor control and acceptable toxicity, while revealing histology-specific volumetric response patterns. These observations should be interpreted with caution, as different inter-fraction intervals were not directly compared in this study.

### 4.2. Radiobiological Rationale

SRS and hfSRS possess fundamentally different radiobiological properties compared with conventional fractionated radiotherapy (RT). Conventional RT involves repeated delivery of low daily doses (1.8–2 Gy), with therapeutic responses explained by the classic “5 Rs”—repair, repopulation, redistribution, reoxygenation, and radiosensitivity—within the framework of the linear-quadratic (LQ) model. In contrast, SRS employs single or few high-dose fractions (≥6–10 Gy), where additional mechanisms beyond DNA double-strand breakage are implicated, including endothelial apoptosis, microvascular collapse, remodeling of the tumor microenvironment, and immune activation [13,14,15,16].

Nevertheless, the LQ-based biologically effective dose (BED) remains a practical tool for quantitative comparison and treatment planning in SRS, despite its known limitations in capturing non-linear biological effects at ablative dose ranges. The LQ model was originally optimized for low-dose fractionation (≤2 Gy per fraction), but its predictive accuracy becomes limited at ablative doses (≥6–10 Gy), where nontraditional mechanisms such as vascular injury and immune stimulation play a major role [14,16,17].

Despite these limitations, numerous SBRT and SRS studies have demonstrated that LQ-BED provides a reasonable approximation of both tumor control probability and normal tissue toxicity, and it remains widely applied in clinical practice due to its practicality and simplicity [18].

In this study, LQ-BED was adopted strictly as a quantitative metric for prescription planning. Interpretation of early volumetric shrinkage and pseudoprogression was intentionally separated from BED-based modeling and instead grounded in high-dose radiobiological mechanisms, including vascular injury and microenvironmental modulation. Although an ablative threshold of ≥8–10 Gy per fraction in CNS hfSRS is theoretically associated with enhanced vascular collapse and immune stimulation [19], standard clinical practice restricts fraction doses to 5–7 Gy to respect organ-at-risk (OAR) constraints and minimize late complications. Thus, an inevitable “dose gap” exists between radiobiological ideals and clinical feasibility, representing a key consideration in CNS radiosurgery design. We did not perform sensitivity analyses using alternative α/β values, which may influence absolute BED estimates but are unlikely to affect the comparative volumetric trends observed.

### 4.3. Fractionation Interval

The choice of a two-week interval between fractions in hypofractionated stereotactic radiosurgery was based on the temporal dynamics of radiation-induced vascular injury, blood–brain barrier (BBB) disruption, and peritumoral edema, rather than on tumor repopulation kinetics. Unlike malignant tumors, benign intracranial tumors demonstrate slow proliferative activity, rendering short inter-fraction intervals designed to suppress repopulation (e.g., 48–72 h) biologically less critical [3].

Following high-dose irradiation, endothelial injury and microvascular dysfunction occur immediately, leading to transient BBB opening and vasogenic edema. Experimental and clinical studies suggest that endothelial repair begins within several days but remains incomplete for up to 2–3 weeks, while astrocytic swelling and vasogenic edema typically peak approximately 2–4 weeks after irradiation [13,14,15,17]. In parallel, radiation-induced immune activation and inflammatory signaling peak within the first week and subsequently decline [19,20,21].

In this context, delivering subsequent fractions at very short intervals (48–72 h) may coincide with ongoing endothelial damage and acute inflammatory responses, potentially amplifying vascular toxicity without additional therapeutic gain in benign tumors. Conversely, excessively long intervals (≥3–4 weeks) risk administering subsequent fractions during the peak edema phase, increasing the likelihood of symptomatic radiation-induced swelling and treatment interruption [15,21].

A fixed two-week interval may represent a balance between competing biological processes [12], including endothelial injury, partial vascular recovery, and the temporal evolution of radiation-induced edema. This timing may allow partial endothelial recovery while delivering the next fraction before maximal edema formation—a concept that may be described as a pre-edema completion strategy. By completing the prescribed fractions prior to the expected peak of vasogenic edema, this approach is hypothesized to allow cumulative biological effects while minimizing acute toxicity [12,15,21].

This approach differs conceptually from both dose-staged radiosurgery, in which fractions are separated by several months to allow edema stabilization, and from weekly or short-interval hypofractionation commonly used in malignant disease [11,12]. Instead, our protocol treats the inter-fraction interval itself as a radiobiological variable, optimized to the unique growth kinetics and toxicity considerations of benign intracranial tumors.

Clinically, this design was associated with acceptable toxicity profiles and distinct volumetric response patterns, including earlier tumor regression in PitNET and more frequent but transient pseudoprogression in vestibular schwannoma, without compromising long-term tumor control. Taken together, these observations indicate that fraction timing—beyond total dose or fraction size—may influence early radiologic response patterns after hfSRS for benign tumors; however, this interpretation should be considered hypothesis-generating, as different inter-fraction intervals were not directly compared in this study.

### 4.4. Fraction Dose Versus Dose-Staged Radiosurgery

hfSRS and dose-staged radiosurgery represent conceptually distinct treatment strategies, despite superficial similarities in fractionated delivery. In hfSRS, fractions are delivered within a relatively short and continuous treatment course, with the intent of exploiting cumulative biological effects of repeated high-dose irradiation while maintaining acceptable organ-at-risk constraints. In contrast, dose-staged radiosurgery separates treatment sessions by several months, primarily to allow resolution of radiation-induced edema or mass effect before additional irradiation [12].

From a radiobiological perspective, dose-staged radiosurgery prioritizes mechanical decompression and toxicity mitigation over temporal synergy between fractions, and thus treats each session as an independent intervention. By contrast, hypofractionated radiosurgery assumes partial biological continuity between fractions, in which vascular injury, microenvironmental modulation, and immune signaling induced by one fraction influence the tissue response to subsequent fractions [13,14,15,17].

The present protocol aligns with the latter paradigm. By delivering 5–7 Gy fractions at fixed two-week intervals, treatment is completed before the peak phase of vasogenic edema, allowing repeated high-dose exposure within a biologically coherent window. This temporal structure is fundamentally different from dose-staged approaches and supports the interpretation of our outcomes—particularly early volumetric regression and transient pseudoprogression—as manifestations of cumulative biological effects rather than staged mechanical debulking.

### 4.5. PitNET

For PitNETs, prior studies and meta-analyses have consistently demonstrated no significant differences between hypofractionated and single-fraction SRS in terms of tumor control, endocrine safety, or complication rates [22,23]. In line with these findings, our matched analysis showed equivalent TCR between hfSRS and sfSRS, but hfSRS was associated with earlier and greater volumetric reduction over time, while maintaining equivalent long-term tumor control. This suggests that in large adenomas or lesions adjacent to the optic apparatus, hfSRS may provide the same long-term control and endocrine safety as single-fraction treatment, while offering the clinical advantage of earlier volume shrinkage [24]. 

### 4.6. Vestibular Schwannoma

In vestibular schwannomas, hfSRS has been widely employed to address dose heterogeneity that may arise with single-fraction SRS in large tumors, where central overdose and peripheral underdosing can occur due to V12 constraints [25,26]. Meta-analyses and large institutional studies have reported broadly comparable rates of tumor control and cranial nerve outcomes between hfSRS and single-fraction SRS, although hearing outcomes remain influenced by baseline imbalance and limited statistical power in many series [25,27,28]. In our cohort, hfSRS and sfSRS achieved equivalent long-term tumor control, while pseudoprogression was more frequent with hfSRS, consistent with known volumetric kinetics in VS [29].

### 4.7. Craniopharyngioma

In our series, craniopharyngiomas demonstrated a relatively greater volumetric reduction compared with other benign tumors. However, existing literature on radiosurgical volumetric dynamics in craniopharyngioma is scarce, and early shrinkage is generally attributed to regression of cystic components rather than solid tumor response [30]. Therefore, our findings should be interpreted cautiously, and further studies are required to validate this observation, given its cystic nature and small sample size in our cohort.

Although meningiomas were not included in the propensity score–matched comparison, tumor control in this subgroup following hypofractionated SRS was favorable and appears broadly consistent with outcomes reported in prior single-fraction radiosurgery series.

### 4.8. Limitations and Further Studies

The present study has several limitations that should be acknowledged. First, the retrospective design inherently introduces the potential for selection bias. Patients treated with hfSRS were often selected based on tumor size, anatomical location, or proximity to organs at risk, which may have resulted in baseline differences compared with the single-fraction cohort. Although propensity score matching was applied for PitNET and vestibular schwannoma to mitigate this bias, residual confounding cannot be completely excluded.

In meningioma, propensity score matching was not performed because anatomical location is intrinsically linked to radiosurgical dose prescription and subsequent clinical outcomes. Skull base meningiomas typically require lower marginal doses due to proximity to the optic apparatus and brainstem, whereas convexity or falcine meningiomas often allow higher prescription doses, resulting in site-dependent differences in tumor control and toxicity profiles. Although stratification by tumor location could theoretically address this confounding, subdividing the meningioma cohort by anatomical site would have resulted in insufficient subgroup sizes, limiting the reliability and interpretability of a matched comparative analysis. Accordingly, meningioma outcomes were analyzed descriptively rather than within a propensity-matched framework.

For PitNETand vestibular schwannoma, the matching models were refined to incorporate tumor-specific variables relevant to treatment selection and radiologic response, including cavernous sinus invasion for PitNET and baseline hearing status and cystic tumor composition for vestibular schwannoma. Despite these efforts, unmeasured factors—such as subtle differences in tumor consistency, vascularity, or prior surgical manipulation—may still have influenced treatment selection and radiologic outcomes. Therefore, functional outcomes, particularly hearing preservation in vestibular schwannoma, should be interpreted with appropriate caution.

Second, inter-fraction volumetric or morphological changes during hypofractionated stereotactic radiosurgery were not systematically quantified. While this limits detailed analysis of short-interval tumor dynamics, treatment decisions and radiosurgical planning in our cohort were not affected by interval changes, and no adaptive replanning was required.

In addition, volumetric measurements were derived from single-observer segmentation, and formal assessment of interobserver variability was not performed. Although this may introduce measurement uncertainty, the use of a consistent observer and standardized workflow across all longitudinal assessments likely mitigated its impact on relative volumetric change over time, which was the primary focus of this study.

Third, although the median follow-up duration of 42 months provides robust evidence for early- to mid-term safety and efficacy, benign intracranial tumors are characterized by indolent growth and the potential for very late recurrence. Consequently, longer follow-up, particularly beyond 10–15 years, is required to establish the long-term durability of tumor control and late toxicity after hypofractionated stereotactic radiosurgery.

Fourth, radiologic response patterns varied substantially by tumor histology. Vestibular schwannomas frequently demonstrated transient post-treatment enlargement consistent with pseudoprogression, whereas meningioma, PitNET, and craniopharyngioma exhibited steadier volumetric trajectories. As a result, simple summary measures of volumetric change may not fully capture tumor-specific temporal response behavior, underscoring the importance of longitudinal interpretation rather than reliance on early post-treatment imaging alone.

Importantly, this study did not directly compare different inter-fraction intervals (e.g., weekly vs. biweekly vs. longer schedules). Therefore, the observed associations between a two-week interval and volumetric response patterns should be interpreted as hypothesis-generating rather than confirmatory.

## 5. Conclusions

This study suggests that hfSRS delivered at a fixed two-week interval can achieve favorable tumor control with acceptable toxicity profiles in selected benign intracranial tumors, particularly PitNET and vestibular schwannoma, for which matched comparative analyses were performed, while demonstrating tumor control rates equivalent to single-fraction SRS and more rapid volumetric reduction. These observations should be interpreted as hypothesis-generating rather than definitive, as different inter-fraction intervals were not directly compared.

Importantly, our study provides robust early- to mid-term outcomes; however, the long-term durability of tumor control in benign intracranial tumors—often requiring follow-up over a decade—remains to be validated. Residual imbalances, such as baseline hearing status, should be addressed in future prospective studies.

## Figures and Tables

**Table 1:** Baseline patient characteristics and treatment parameters.

Variables (*n* = 126)	Values
Male, *n* (%)	40 (31.7)
Age, median (IQR)	51 (39–61)
Tumor types, *n* (%)	
Meningioma	32 (25.4)
NF-PitNET	34 (27.0)
Schwannoma	49 (38.9)
Craniopharyngioma	11 (8.7)
Tumor locations, *n* (%)	
Orbital cavity	5 (3.9)
Parasellar region	66 (52.4)
Cerebellopontine angle	51 (40.5)
Others	4 (3.2)
On HRT before irradiation, *n* (%)	26 (20.6)
Radiosurgery as primary treatment, *n* (%)	63 (50.0)
Previous irradiation, *n* (%)	12 (9.5)
Radiosurgical variables	
Frameless:frame-based	114:12
Tumor volume, median cm^3^ (range)	2.3 (0.4–25.85)
Intention for functional preservation, *n* (%)	
Vision	71 (56.3)
Hearing	48 (38.1)
Marginal dose per tumor type When converted to a single fraction SRS, median Gy (range)	
Meningioma	13 (12–15)
NF-PitNET	14 (13.5–15)
Schwannoma	13 (12–13)
Craniopharyngioma	14 (14–15)

Note: HRT, hormone replacement therapy; IQR, interquartile range; NF-PitNET, non-functioning pituitary neuroendocrine tumors.

**Table 2:** Overall radiological and clinical outcomes of hypofractionated stereotactic radiosurgery (hfSRS).

Radiological Outcomes (*n* = 126)	
Follow-up, median months (IQR)	42 (36–55)
TCR, *n* (%)	124 (98.4)
Percentage volume change per month, median (IQR)	−0.64 (−2.01, 0.37)
Meningioma	−0.59 (−0.84, −0.13)
PA	−0.76 (−2.52, −0.01)
Schwannoma	−0.21 (−2.03, 1.48)
Craniopharyngioma	−2.38 (−3.72, −1.89)
Hydrocephalus, *n* (%)	2 (1.5)
Radiation-induced edema, *n* (%)	3 (2.4)
Vascular compromise, *n* (%)	3 (2.3)
Clinical outcomes (*n* = 126)	
Follow-up, median months (IQR)	42 (36–60)
Complications per cases at risk, *n* (%)	
Facial palsy aggravation	1/51 (2.0)
Functional hearing loss	16/39 (41.0)
GR I → GR III or higher	5/23 (21.7)
GR II → GR III or higher	11/16 (68.8)
RION, *n* (%)	2/71 (2.8)
New onset hypopituitarism, *n* (%)	2/40 (5.0)

Note: IQR, interquartile range; TCR, Tumor control rate; GR, Gardner–Robertson classification; RION, radiation-induced optic neuropathy.

**Table 3:** Patient outcomes for PA and VS after 1:1 propensity score matching.

Variables	hfSRS	sfSRS	*p* Value
PitNET (*n* = 27)			
Tumor control rate, *n* (%)	26/27 (96.3)	27/27 (100)	>0.99
Percentage volume change per month, median (IQR)	−0.79 (−1.83, 0.48)	−0.14 (−0.54, 0.28)	0.029
RION, *n* (%)	1/27 (3.7)	2/27 (7.4)	0.55
New hypopituitarism, *n* (%)	1/27 (3.7)	2/27 (7.4)	0.55
VS (*n* = 41)			
Tumor control rate, *n* (%)	40/41 (97.6)	41/41 (100)	>0.99
Percentage volume change from baseline at 0–12 months, median (IQR)	+1.43 (−0.31, 3.20)	+0.14 (−0.20, 0.48)	0.17
Percentage volume change from baseline at 0–36 months, median (IQR)	−0.23 (−2.03, 1.42)	−0.13 (−0.73, 1.35)	0.49
Pseudoprogression, *n* (%)	17/41 (41.5)	8/41 (19.5)	0.03
GR I prior to SRS, *n* (%)	18/41 (43.9)	11/41 (26.8)	0.11
Serviceable hearing prior to SRS, *n* (%)	30/41 (73.2)	23/41 (56.1)	0.10
Functional hearing loss, *n* (%)	12/30 (40.0)	14/23 (60.9)	0.17

Note: GR, Gardner-Robertson Classification; hfSRS, hypofractionated stereotactic radiosurgery; sfSRS, single fraction stereotactic radiosurgery; PitNET, pituitary neuroendocrine tumors; RION, radiation-induced optic neuropathy; VS, vestibular schwannoma.

## Data Availability

The data presented in this study are available from the corresponding authors upon reasonable request.

## Supplementary Materials

The following supporting information can be downloaded at: https://www.mdpi.com/article/10.3390/cancers18040617/s1, Figure S1: Flow diagram of patient selection, Table S1: Dose conversion of representative marginal doses and OAR constraints (LQ model), Table S2: Basic characteristics of pituitary neuroendocrine tumor patients, Table S3: Basic characteristics of vestibular schwannoma patients.
