# Supplementary material for: Two-Week Interval Hypofractionated Stereotactic Radiosurgery for Benign Intracranial Tumors: Volumetric Kinetics and Radiobiological Rationale
Source: Cancers (Basel). 2026 Feb 13;18(4):617. doi: 10.3390/cancers18040617 (PMC12939977; doi:10.3390/cancers18040617)
Supplement: Supplementary file 1 [file cancers-18-00617-s001.zip › Supplementary(260114).pdf]

Supplementary Figure S1. Flow diagram of patient selection

Note ; Between March 2016 and February 2022, 157 patients with benign intracranial tumors underwent hypofractionated stereotactic radiosurgery (hfSRS). Thirty-one patients were excluded: 19 with functioning pituitary adenoma, 3 with hemangioma, 2 with pituitary oncocytoma, and 7 with insufficient follow-up (<36 months or loss of radiological follow-up). The final study cohort included 126 patients: 32 meningiomas, 34 non-functioning pituitary adenomas (NFPA), 49 vestibular schwannomas (VS), and 11 craniopharyngiomas.

Supplementary Table S1. Dose conversion of representative marginal doses and OAR constraints (LQ model)

Single-fraction marginal dose (Gy)	BED (Gy)	3 Fraction schedule				5-Fraction schedule			
		Dose per fraction		OAR dose constraints		Dose per fraction		OAR dose constraints	
		Calculated	Applied	AOP	Cochlea	Calculated	Applied	AOP	Cochlea
13.0	69.33	6.96	7.00	5.40	3.12	5.12	5.10	4.00	2.26
14.0	79.33	7.53	7.50			5.56	5.50 or 5.60		

Note: BED was calculated using the linear-quadratic (LQ) model: BED = nd(1 + d/[α/β]), where n represents the number of fractions, d the dose per fraction (Gy), and α/β the tissue-specific radiosensitivity parameter (assumed as 3 for tumor and 2 for OAR). Applied dose values reflect rounding during physical planning (increments of 0.1–0.25–0.5 Gy).

Supplementary Table S2. Basic characteristics of PA patients

	**Unmatched cohorts**		**Matched cohorts**			
	HFSRS (n=34)	Single (n=164)	HFSRS (n=27)	Single (n=27)	p- value	SMD
**Age, years(median [IQR])**	44 [32–56]	51[44–62]	47[36–56]	50[37–58]	0.36	0.07
**Sex (male), n (%)**	11,32.4	48,29.3	8, 29.6	10,31.5	0.51	0.05
**Baseline tumor volume, cm_3 (median [IQR])**	2.63 [0.41–4.34]	2.15[0.15–4.90]	2.63[0.49–4.34]	2.51 [0.59–4.63]	0.62	0.04
**Marginal dose, Gy (median [IQR])**	14 [14–15]	14 [14–16]	14 [14–15]	14 [14–15]	0.66	0.00
**Knosp grade ≥3, n (%)**	28 (82.4)	96(58.5)	22(81.5)	21(77.8)	0.74	0.08

Note: IQR, interquartile range; PA, pituitary adenoma

Supplementary Table S3. Basic characteristics of VS patients

	Unmatched cohorts		Matched cohorts			
	HFSRS (n = 46)	Single (n = 297)	HFSRS (n = 41)	Single (n = 41)	p-value	SMD
**Age, years (median [IQR])**	54.5 [46–62]	54.0 [44–64]	54.0 [47–62]	54.0 [45–62]	*0.48*	0.00
**Sex (male), n (%)**	15 (32.6)	130 (43.8)	14 (34.1)	16 (39.0)	*0.82*	0.09
**Baseline tumor volume, cm_3 (median [IQR])**	11.93 [0.26–24.13]	13.40 [0.53–26.79]	12.97 [2.01–24.13]	13.05 [0.53–24.41]	*0.63*	0.00
**Marginal dose, Gy (median [IQR])**	13 [12–13]	13 [12–15]	13 [12-13]	13 [12–13]	> *0.99*	0.00
**Serviceable hearing at baseline, n (%)**	33 (71.7)	161 (54.2)	30 (73.2)	23 (56.1)	*0.10*	0.16
**Cystic tumor component, n (%)**	16 (34.8)	89 (30.0)	14 (34.1)	13 (31.7)	*0.82*	0.05
**Koos grade III–IV, n (%)**	26 (56.5)	145 (48.8)	22 (53.7)	20 (48.8)	*0.66*	0.08

Note: IQR, interquartile range; VS, vestibular schwannoma; SMD, standard mean difference
