# Supplementary figures and images for: Two-Week Interval Hypofractionated Stereotactic Radiosurgery for Benign Intracranial Tumors: Volumetric Kinetics and Radiobiological Rationale
Source: Cancers (Basel). 2026 Feb 13;18(4):617. doi: 10.3390/cancers18040617 (PMC12939977; doi:10.3390/cancers18040617)

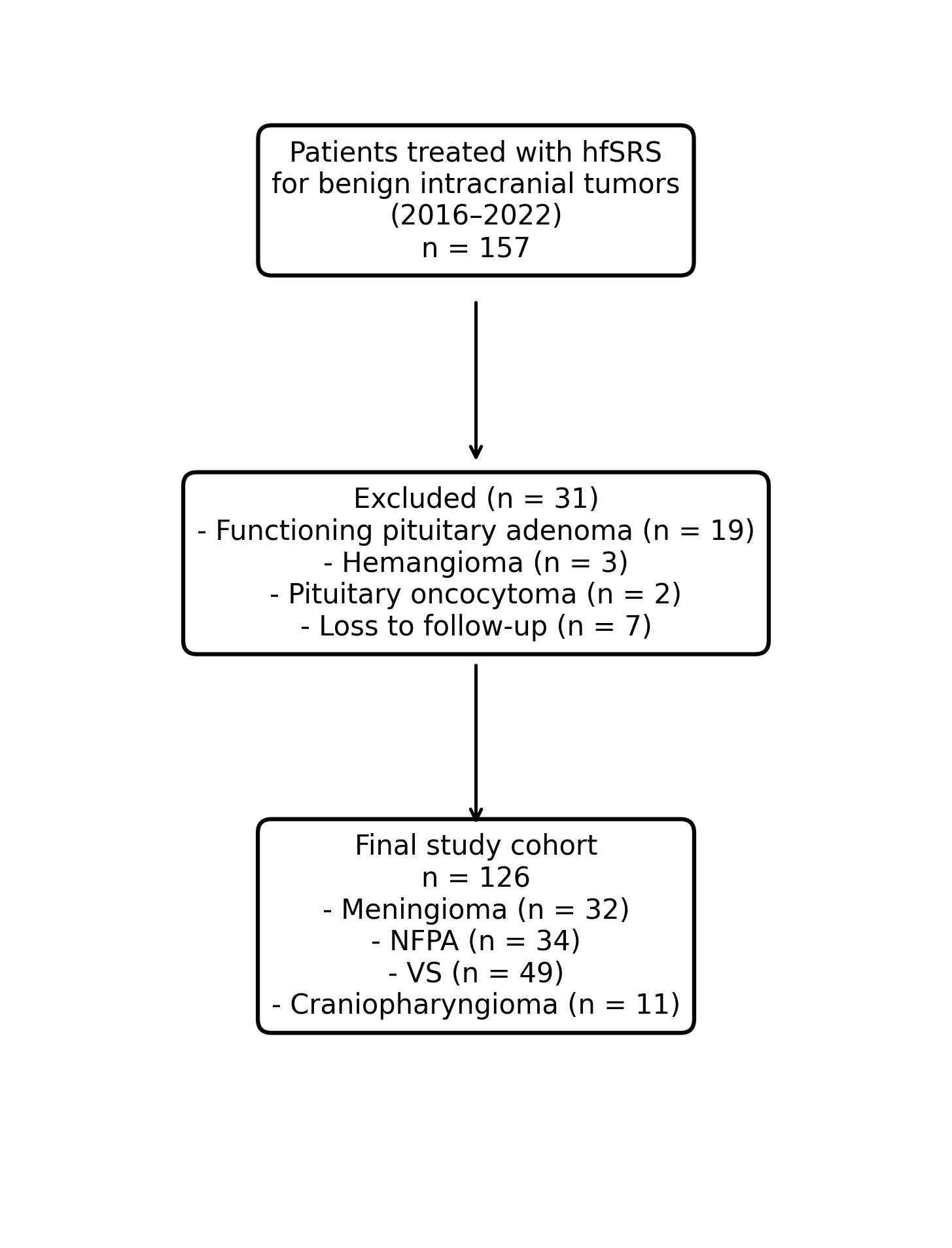

Supplement: Supplementary file 1 [file cancers-18-00617-s001.zip › FigureS1.png]
